# Microelectrode Sensor for Real-Time Measurements of Nitrite in the Living Brain, in the Presence of Ascorbate

**DOI:** 10.3390/bios11080277

**Published:** 2021-08-17

**Authors:** Tiago Monteiro, Cândida Dias, Cátia F. Lourenço, Ana Ledo, Rui M. Barbosa, M. Gabriela Almeida

**Affiliations:** 1 UCIBIO—Applied Molecular Biosciences Unit, REQUIMTE—Rede de Química e Tecnologia, Faculdade de Ciências e Tecnologia, Universidade NOVA de Lisboa, 2829-516 Monte de Caparica, Portugal; tc.monteiro@campus.fct.unl.pt; 2 Center for Neuroscience and Cell Biology, University of Coimbra, 3004-504 Coimbra, Portugal; candidamndias@gmail.com (C.D.); catiaflmarques@gmail.com (C.F.L.); analedo@cnc.uc.pt (A.L.); 3 Health Sciences Campus, Faculty of Pharmacy, University of Coimbra, Azinhaga de Santa Comba, 3000-548 Coimbra, Portugal; 4 Centro de Investigação Interdisciplinar Egas Moniz (CiiEM), Instituto Universitário Egas Moniz, Campus Universitário, Quinta da Granja, 2829-511 Monte de Caparica, Portugal

**Keywords:** carbon fiber microelectrodes, fast-scan cyclic voltammetry, nitrite, hippocampus

## Abstract

The impaired blood flow to the brain causes a decrease in the supply of oxygen that can result in cerebral ischemia; if the blood flow is not restored quickly, neuronal injury or death will occur. Under hypoxic conditions, the production of nitric oxide (^●^NO), via the classical L-arginine–^●^NO synthase pathway, is reduced, which can compromise ^●^NO-dependent vasodilation. However, the alternative nitrite (NO_2_^−^) reduction to ^●^NO, under neuronal hypoxia and ischemia conditions, has been viewed as an in vivo storage pool of ^●^NO, complementing its enzymatic synthesis. Brain research is thus demanding suitable tools to probe nitrite’s temporal and spatial dynamics in vivo. In this work, we propose a new method for the real-time measurement of nitrite concentration in the brain extracellular space, using fast-scan cyclic voltammetry (FSCV) and carbon microfiber electrodes as sensing probes. In this way, nitrite was detected anodically and in vitro, in the 5–500 µM range, in the presence of increasing physiological concentrations of ascorbate (100–500 µM). These sensors were then tested for real-time and in vivo recordings in the anesthetized rat hippocampus; using fast electrochemical techniques, local and reproducible transients of nitrite oxidation signals were observed, upon pressure ejection of an exogenous nitrite solution into the brain tissue. Nitrite microsensors are thus a valuable tool for investigating the role of this inorganic anion in brain redox signaling.

## 1. Introduction

The classical enzymatic production of the free radical messenger nitric oxide (^•^NO) in vivo, is carried out by the enzyme nitric oxide synthase (NOS), which has the following three isoforms: neuronal (nNOS), endothelial (eNOS), and inducible (iNOS) [1,2,3]. This process occurs in a two-step reaction, where L-arginine is oxidized, requiring a reduction in oxygen [4].

Impaired blood flow to the brain causes a decrease in the supply of oxygen and glucose, which are necessary to maintain the energetic metabolism of glial and neuronal cells [5,6]. This results in cerebral ischemia and, if the blood flow is not restored quickly, neuronal injury or death will occur [2,5]. Upon hypoxic conditions, the (oxygen-dependent) enzymatic production of ^●^NO is highly attenuated and, consequently, this can be translated into a subsequent compromised ^●^NO-dependent vasodilation [7]. In this scenario, nitrite may contribute to the maintenance of ^●^NO levels, since the tissue-accumulated nitrite has the potential to reduce back to ^●^NO under hypoxia [8]. The nitrite concentration in tissues has been estimated to be in the 0.1–10 µM range [9,10], but it can be easily increased with diet, through the ingestion of nitrate-rich foods [8]. Nitrate accumulates in the oral cavity, and commensal facultative anaerobic bacteria reduce it to nitrite, by the action of nitrate reductases [11]. Because the classical L-arginine–NOS pathway is oxygen-dependent, and the nitrate–nitrite–^●^NO pathway is enhanced under hypoxic conditions, the latter can be viewed as a backup system to ensure that there is sufficient vasodilator formation when the oxygen supply is compromised [8,12,13].

Topical administration of 1 µM nitrite to the brain (somatosensory cortex) has been demonstrated to promote the recovery of cerebral blood flow after inhibition of NOS activity, showing that the brain tissue can use nitrite as an alternative source of ^●^NO [14]. However, no information was obtained about the mechanism through which this conversion occurs. Enzymes such as deoxyhemoglobin and xanthine oxidase, are known to recycle nitrite back to ^●^NO in low oxygen tension and acidic conditions [8,12,13]. Alternatively, a simpler mechanism was proposed over 20 years ago, describing the direct and non-enzymatic conversion of nitrite to ^●^NO by ascorbic acid [15]. This hypothesis proposed that upon glutamatergic stimulation, ascorbate is released into the brain extracellular space [15,16]. The pH drops with the decrease in the local oxygen levels, and, under these conditions, ascorbate readily reduces nitrite to ^●^NO, which, upon diffusion to the smooth muscle cells of nearby vessels, may induce local vasodilation.

Ascorbate and ^●^NO were shown to be temporarily and spatially related in vivo, in a quantitative fashion, upon glutamatergic stimulus in the hippocampus of animal models [17]. These observations, coupled with the onset reduction in nitrite (in vitro) in the presence of ascorbate, when media pH was lowered from 7.4 to 6.5 [7], are compelling arguments that are in favor of the nitrite–^●^NO acidic reduction pathway.

In this context, the development of analytical tools for real-time and in vivo measurements of nitrite concentration dynamics is of great interest. It is also important to ascertain if ascorbate, which is present in high and varying concentrations in the brain, does not interfere with measurements (ascorbate is an electroactive species, and its oxidation products could affect both faradaic and non-faradaic currents).

A major obstacle to the real-time study of the extracellular chemical composition derives from the limited spatial and temporal resolutions of the more frequently used monitoring techniques (e.g., microdialysis sampling, spectroscopic techniques) versus the dynamics of chemical signaling and the structures of interest in the brain [18,19]. Furthermore, since measurements performed directly in live tissue are invasive by nature, the insertion of large probes (e.g., microdialysis probe with a 200 µm diameter) will potentially result in tissue damage [19].

Alternatively, electrochemical methods differ from dialysis, in the way that compounds can be measured in situ and in real-time, allowing the study of the dynamics affecting the in vivo concentrations [20,21,22]. Also, the employment of microelectrodes offers high sensitivity and spatial resolution (typical dimensions: length 100–200 µm; diameter 10–30 µm). Carbon fiber microelectrodes (CFMs), in particular, can be constructed with single-digit micrometer dimensions, which are much smaller than typical microdialysis probes. They can be placed within micrometer distances of neuronal terminals, with the added bonus of minimal tissue damage and high biocompatibility [23,24,25]. CFMs have thus been extensively used over the last four decades, as an important analytical tool to investigate brain function in vivo, ex vivo, and at the single-cell level. The use of CFMs has improved electrochemical recordings, as it allows the application of faster scan rates due to the small surface area, minimizing the effects of background charge and ohmic drop [26,27]. Moreover, hand-made fabrication of bare CFMs has many advantages, including low cost, ease of fabrication, and customizability. The selectivity of CFMs may be increased through the use of coatings and polymers. However, the process is time consuming, expensive, and often leads to poor reproducibility. Additionally, during experiments, the coatings may fail, due to cracking, peeling, or chemical degradation [28].

The combination of CFMs with fast data acquisition methods provides the temporal resolution that is required to evaluate the kinetics of many different neurochemical species in the living brain. This is the case of fast-scan cyclic voltammetry (FSCV), in which the current is recorded as a function of potential that is swept at very high rates (over 300 V s^−1^), thus enabling the real-time detection of neurotransmitters on the sub-second time scale. In spite of the high scan rates of the potential waveform, this technique does not affect neuronal activity [18,19,27,29]. Another advantage of FSCV in association with CFMs, over other electrochemical methods for in vivo and ex vivo recordings, relies on the selectivity of detection, i.e., ability to distinguish electroactive species based on their redox potentials, without the need of further modification of microelectrodes with membrane coatings and other layers. In this way, the collected data provide information on the signature of the redox species, and on their dynamics in the surrounding media [27,29,30,31]. High-speed, highly localized measurements of dopamine [32,33] and ^●^NO [34,35,36], for example, have been extensively obtained in the brain extracellular space, with unparalleled spatiotemporal resolution.

To the best of the authors’ knowledge, dynamic in vivo measurements of nitrite have never been reported. Therefore, the present work addresses a novel sensing strategy for the detection and monitoring of nitrite in vitro and in vivo, in the brain tissue. Homemade carbon fiber microelectrodes (CFM) were used as sensing platforms, coupled with fast electrochemical techniques. The microsensor was tested for the real-time detection of nitrite in the hippocampus of an anesthetized rat.

## 2. Materials and Methods

### 2.1. Reagents and Solutions

Acetone was obtained from Sigma-Aldrich (St. Louis, MO, USA). Sodium ascorbate was obtained from Fluka. Dibasic sodium phosphate, monobasic sodium phosphate and sodium nitrite were obtained from Panreac. Silver conductive paint was purchased from RS Components. All reagents were of analytical grade. Solutions were prepared with deionized water (≥18 MΩ cm) from a Millipore Milli-Q purification system.

### 2.2. Carbon Fiber Microelectrode Fabrication and Calibration

Carbon fiber microelectrodes were fabricated as described in reference [36]. Briefly, single carbon fibers (*ø* = 7 µm, Goodfellow, Huntingdon, UK) were inserted into borosilicate glass capillaries (1.16 mm i.d. × 2.0 mm o.d.; Harvard Apparatus Ltd., Cambourne, UK), previously filled with acetone. After the evaporation of the solvent at room temperature, the capillary containing the carbon fiber was separated in half using a vertical puller (Harvard Apparatus Ltd., Cambourne, UK), with the exposed fiber being cut. Afterwards, the exposed carbon tip was trimmed, under the optical microscope, to obtain a length between 150 and 250 µm. The electrical contact was made by injecting a conductive silver paint into the capillary, followed by the insertion of a copper wire. A small drop of cyanoacrylate glue was used over the glass opening from where the copper wire protruded, to hold the latter in place. Finally, a protective rubber sleeve was placed over the glued junction and heated, thus producing a protective cap.

The produced microelectrodes were tested for general recording properties in 50 mM phosphate buffer (pH 7.4), with 100 mM NaCl supporting electrolyte. This was carried out by fast-scan cyclic voltammetry (FSCV, potentiostat from Ensman Instruments, Bloomington, IN, USA), by sweeping the potential between −0.4 and 1.4 V (initially in the anodic direction), for 30 s, at a 300 V s^−1^ scan rate. A two-electrode configuration was used, with a Ag/AgCl 3 M KCl reference electrode from BASi (West Lafayette, IN, USA). The selection of proper microelectrodes for recording was made based on the following criteria: stable background current and sharp transients at reversal potentials [36].

Microelectrode calibration was conducted by spiking the supporting electrolyte with nitrite and ascorbate standard solutions (50–500 µM) and recording the FSCV voltammograms between −0.4 and 1.4 V, at a 300 V s^−1^ scan rate and with display rate of 10 Hz, using the data acquisition software Scope v4.1.4 (eDAQ). Nitrite calibration (5–500 µM) in the presence of ascorbate (100–500 µM) was performed in the same conditions but sampling the voltammograms at 1.1 V (vs. reference system) using the data acquisition software Chart v5.5.20 (eDAQ).

The background current of all FSCV profiles was subtracted using OriginPro 2016 v9.3.226 (OriginLab Corporation, Northampton, MA, USA) and smoothed using the 10-point FFT filter. The QSoas v1.0 (CNRS, Marseille, France) [37] non-catalytic baseline algorithm was used to baseline correct the smoothed FSCV profiles.

The obtained calibration curves were used to calculate the sensitivity (slope) and the LOD, which was taken to be *3S_a_/m*, where *S_a_* was the standard deviation of the *y*-intercept and *m* the slope of the calibration curve.

### 2.3. In Vivo Measurements of Nitrite and Ascorbate Using the Microelectrode Sensor

The in vivo studies were performed on an adult male Wistar rat (300 g; Charles River Laboratories). Animals were housed in pairs in the local vivarium in a 12 h light/dark cycle, at a temperature of 22–24 °C, relative humidity of 45–65%, air exchange rate of 15 times per hour, and with food and water available ad libitum.

The experimental setup used for electrochemical detection of nitrite, and ascorbate in vivo was analogous to previous studies [38]. Briefly, the animal was anesthetized with urethane (1.25 g kg^−1^) via intraperitoneal injection, placed in a stereotaxic frame (Stoelting, Wood Dale, IL, USA), body temperature was maintained at 37 °C and the skull exposed. A craniotomy was made in the region of interest with removal of the meninges to expose the brain surface. Another hole was drilled in a site remote from the recording area for the insertion (*ca*. 3 mm) of a homemade Ag/AgCl pseudo-reference electrode in the subdural space. The latter was constructed by oxidizing the exposed tip of a silver wire (*ø* = 200 μm, Science Products, Hofheim, Germany) in a 1 M HCl solution saturated with NaCl [39].

A 7 µm o.d. bare carbon fiber microelectrode, previously calibrated for both nitrite and ascorbate in 50 mM phosphate buffer (pH 7.4), with 100 mM NaCl, was coupled with a glass micropipette (tip *ø* = 10–15 µm) using sticky wax (softened by flame), according to a configuration in which tips separation was 250 µm. The micropipette was then filled using a syringe fitted with a flexible microfilament (MicroFil, World Precision Instruments, Hitchin, UK). The solutions had either 5 mM nitrite or 50 mM ascorbate in saline solution (0.9% NaCl); the pH was set at 7.4.

The microelectrode/micropipette array was inserted into the hippocampus using a stereotaxic frame, according to coordinates calculated based on rat brain atlas of Paxinos and Watson (2007) [40]. The coordinates used to record the analytes dynamics in the CA1 subregion of the hippocampus (calculated from bregma) were the following: AP (antero-posterior) −4.0 mm; ML (medio-lateral) −2.1 mm; DV (dorso-ventral) −2.3 mm (*cf*. Figure S2).

Following the insertion of the array in the brain, the baseline current was allowed to stabilize (*c.a.* 30 min). Afterwards, the standard solution was pressure ejected (nL range) from the micropipette using a Picospritzer III (Parker Hannifin Corp., General Valve Operation, Hollis, NH, USA). The applied volume was monitored using a stereomicroscope fitted with a reticule. FSCV profiles were recorded at a 300 V s^−1^ scan rate, between −0.4 and 1.4 V, and sampled at hold potential 1.1 V for nitrite and 0.2 V for ascorbate, with a 10 Hz data acquisition rate.

## 3. Results and Discussion

### 3.1. Carbon Fiber Microelectrode—Nitrite and Ascorbate In Vitro Calibrations

**Figure 1:**
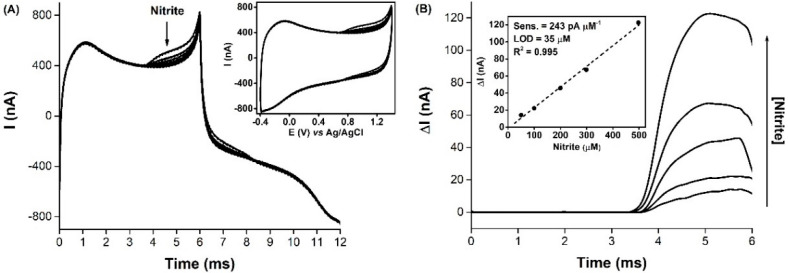
In vitro response of the carbon fiber microelectrode to increasing nitrite concentrations, as determined by FSCV. (**A**) FSCV profiles for increasing nitrite concentrations (50–500 µM); inset shows the corresponding cyclic voltammograms. (**B**) Background subtracted anodic traces, with 10-point FFT filter and baseline correction. Inset: linear correlation between nitrite concentration and anodic current determined at 1.1 V vs. Ag/AgCl (5 ms). Measurements were performed in 50 mM phosphate buffer (pH 7.4), with 100 mM NaCl, at a scan rate of 300 V s^−1^.

The in vitro electrochemical detection of nitrite and ascorbate, using a carbon fiber microelectrode (7 µm o.d.) as the working electrode, was carried out by spiking the buffered supporting electrolyte with standard solutions of each analyte and recording the respective FSCV profiles. The voltammograms were then sampled at specific potentials for the respective oxidations on the solid electrode surface, with the proportional current changes being plotted against the known analyte concentrations. For nitrite (Figure 1A), the consecutive addition of stock solutions to the buffered media was accompanied by an increase in the anodic current, with a wide oxidation wave starting at about 0.65 V vs. Ag/AgCl (*c.a.* 3.5 ms, for a scan rate of 300 V s^−1^ and a start potential of −0.4 V vs. Ag/AgCl) and reaching a maximum of around 1.1 V (*c.a.* 5 ms). This behavior is in agreement with other previously reported unmodified carbon fiber microsensors for nitrite detection [41,42], where it was proposed that nitrite was being oxidized to nitrate, according to Equation (1) [42,43].

(1) $$(1)\;{\mathrm{NO}}_{2}^{\;\;-}\;\to {\;\mathrm{NO}}_{2}^{\;}{+e}^{-}\;(2)\;{2\mathrm{NO}}_{2}^{\;}{+H}_{2}O\;\to {\;\mathrm{NO}}_{2}^{\;\;-}{+\mathrm{NO}}_{3}^{\;\;-}{+2H}^{+}$$

To better observe this electrochemical behavior, the voltammograms were subject to a signal processing procedure [18,27]. First, the background current was subtracted from all the voltammograms, to eliminate the contribution of the capacitive current. The resulting anodic traces were then smoothed, and baseline corrected (Figure 1B). A linear correlation between the anodic current response (at 1.1 V vs. Ag/AgCl) and nitrite concentration, was obtained in the 50–500 µM range, with a sensitivity of 243 pA µM^−1^ and a LOD of 35 µM (Figure 1B, inset). It should be noted that the tested nitrite concentration range was much higher than the expected physiological levels in the brain tissue (0.5 to 27 µM, as obtained by microdialysis) [44,45,46,47,48,49]. The selected concentrations were thus chosen as a preliminary working range, to detect the proper anodic signals. On another note, it should also be stated that the potential at which nitrite oxidation was observed is susceptible to interference by ^●^NO. For a bare carbon fiber electrode, the oxidation peak for ^●^NO occurs at 1.1 V vs. Ag/AgCl and is accompanied by a reduction peak at –0.4 V, while nitrite is not electroactive at that potential [35,50]. This difference in the profiles is key to distinguish between the two, so in vivo measurements using the microsensor approach must be made with this consideration in mind.

**Figure 2:**
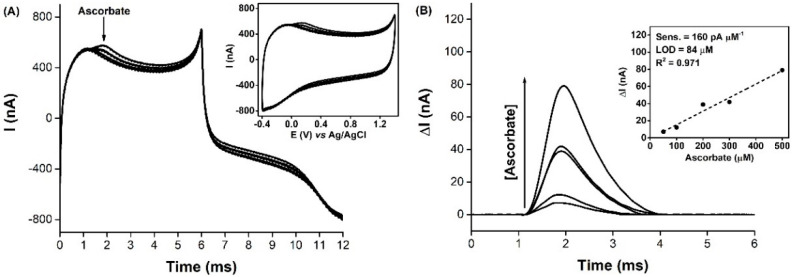
In vitro response of the carbon fiber microelectrode to increasing ascorbate concentrations, as determined by FSCV. (**A**) FSCV profiles for increasing ascorbate concentrations (50–500 µM); inset shows the corresponding cyclic voltammograms. (**B**) Background subtracted anodic traces, with 10-point FFT filter and baseline correction. Inset: linear correlation between ascorbate concentration and anodic current determined at 0.2 V vs. Ag/AgCl (2 ms). Measurements were performed in 50 mM phosphate buffer (pH 7.4), with 100 mM NaCl, at a scan rate of 300 V s^−1^.

As for nitrite, a similar FSCV recording and signal treatment procedure was carried out for ascorbate, and the resulting voltammograms and calibration curve are presented in Figure 2. The anodic peak of ascorbate occurred at around 0.2 V vs. Ag/AgCl (*c.a.* 2 ms), which is in agreement with the previously reported bare carbon microelectrodes [51] and macroelectrodes [52]. However, the raw oxidation signals (Figure 2A) were ill-defined, which could be attributed to a slow electron-transfer process of ascorbate at the surface of the electrode, possibly due to fouling caused by the deposition of the oxidation product 2,3-diketogulonic acid [53,54]. At the electrode surface, the two-electron oxidation of ascorbate produces dehydroascorbate, which is irreversibly hydrolyzed to 2,3-diketogulonic acid [55], a non-electroactive product that readily adsorbs onto the surface of the electrode [54]. Nonetheless, the current increased linearly in the concentration range tested (Figure 2B), with the microsensor having displayed a sensitivity, towards ascorbate, of 160 pA µM^−1^ and a LOD of 84 µM, demonstrating that, compared to nitrite, the carbon fiber microelectrode was less sensitive to ascorbate oxidation. Also, the oxidation potentials of ascorbate and nitrite were sufficiently separated, for the two molecules to be distinguished by observation of the FSCV profile.

**Figure 3:**
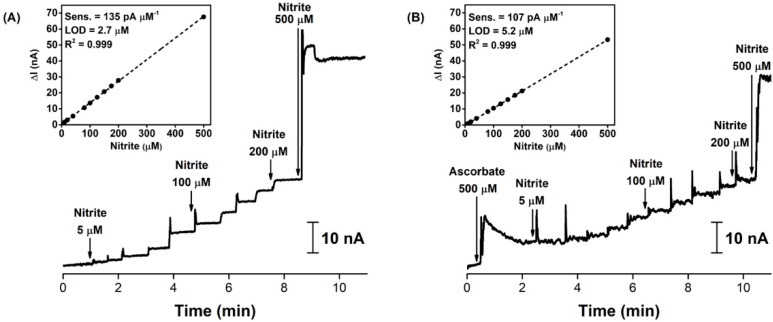
In vitro detection of nitrite using a carbon fiber microelectrode and current sampling by FSCV at a hold potential of 1.1 V. Nitrite standard solution was consecutively added at the time indicated by each arrow, in the 5–500 µM concentration range to a 50 mM phosphate buffer (pH 7.4), 100 mM NaCl solution, (**A**) without and (**B**) in the presence of 500 µM ascorbate. Insets: nitrite calibration curves.

Additional microelectrodes were tested in vitro, for the detection of nitrite in the presence of ascorbate, at the concentrations found in the cerebral extracellular space (200–400 µM, in rat model) [56,57]. Therefore, a series of nitrite standard additions were performed in pH 7.4 buffer media, with 0, 100, 200 and 500 µM ascorbate in solution, and the current was sampled at 1.1 V vs. Ag/AgCl, from consecutive FSCV profiles. Figure 3 shows the current variation, with successive additions of nitrite, either with no ascorbate present in the background (Figure 3A), or with 500 µM ascorbate (Figure 3B). The calculated analytical parameters of sensitivity, linear range and LOD for each condition are presented in Table 1.

Overall, the microelectrode was able to detect all the concentrations of nitrite added, showing a good linear response in the range 5–500 µM, which was kept unchanged, with the sensitivity dropping around 8%, 12% and 21% when 100, 200 and 500 µM ascorbate was added to the media, respectively. Also, the determined LOD values fluctuated, but were kept in the same order of magnitude.

The approach of sampling the current at a fixed potential, rather than recording the individual FSCV profiles, was shown to be more straightforward and simpler to operate, without the need of signal processing that the individual recording of FSCV profiles required. It should be noted that, upon the addition of ascorbate to the buffered media, the baseline current increased and took about 2 min to stabilize. Although the oxidation peak for ascorbate (0.2 V vs. Ag/AgCl) was well below the chosen sampling potential (1.1 V vs. Ag/AgCl), the anodic current of the FSCV profile also increased for higher potentials when compared to the background current, as evidenced in Figure 2A. This explains the initial increase in the baseline current that is observed in Figure 3B, upon the addition of 500 µM ascorbate.

These results were of great relevance, since in vivo nitrite measurements in the brain extracellular space are performed in an ascorbate-rich environment and the sensor has been shown to be able to detect a slight increase in the anodic current (0.3 nA) when 5 µM nitrite was added to the buffering media containing 100-fold more ascorbate.

### 3.2. In Vivo Nitrite and Ascorbate Measurements

Measurements of exogenously applied nitrite and ascorbate were performed in the brain extracellular space of an anesthetized rat. The bare carbon fiber microsensor (coupled with a glass micropipette) was inserted into the hippocampus CA1 subregion, and standard solutions of either nitrite (5 mM) or ascorbate (50 mM) were locally pressure ejected in small volumes (nL range). The current variation was monitored by FSCV and sampling at 1.1 V and 0.2 V vs. pseudo Ag/AgCl, for nitrite and ascorbate oxidation, respectively.

**Figure 4:**
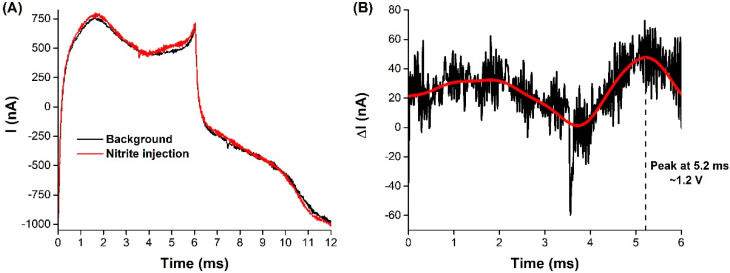
In vivo detection of exogenous nitrite applied locally in the cerebral extracellular space of the rat hippocampus. (**A**) FSCV profiles of background current (black trace) and local bolus injection of 625 pmol of nitrite (red trace). (**B**) Profile obtained from the difference between nitrite addition and background current profiles, raw (black) and 60-points FFT smoothed (red) traces. Electrochemical measurements were performed at 300 V s^−1^ scan rate, from −0.4 to 1.4 V vs. pseudo Ag/AgCl.

The recorded FSCV profiles, before and after the bolus injection of 625 pmol of nitrite, are shown in Figure 4A. There were no detectable oxidation or reduction signals in the background current profile. Upon the injection of the nitrite solution in the brain tissue, close to the microelectrode (distance of 250 µm), a small increase in the anodic current was briefly observed in the FSCV profile, between 4 and 6 ms, reaching a maximum value around 1.2 V vs. pseudo Ag/AgCl (5.2 ms, with a 300 V s^−1^ scan rate and a starting potential of –0.4 V at *t*_0_ min)—see Figure 4B. This result was in agreement with the oxidation signal detected in the in vitro nitrite calibrations performed in Section 3.1, where the oxidation peak was obtained at about 1.1 V vs. Ag/AgCl. The positive potential shift of 100 mV could be due to the difference between the buffer used in the in vitro assay and the live brain matrix, the difference between the reference and pseudo-reference electrodes used in the respective assays, or a combination of both. Nonetheless, the similarity of both signals is a compelling evidence that the oxidation of nitrite was being detected on the surface of the microelectrode.

However, the current measurements suffered from electrical noise interference and the background profile kept shifting during the recordings. For these reasons, the background subtraction method, demonstrated in Section 3.1, was not used in the in vivo experiments. Thus, since the in vivo nitrite FSCV profile matched the one presented in the in vitro calibration, attesting that the microsensor was measuring localized changes in the nitrite levels, the in vivo experiments were further carried out by sampling the potential at 1.1 V, the potential at which the microelectrode had previously been calibrated for nitrite detection in buffered media.

**Figure 5:**
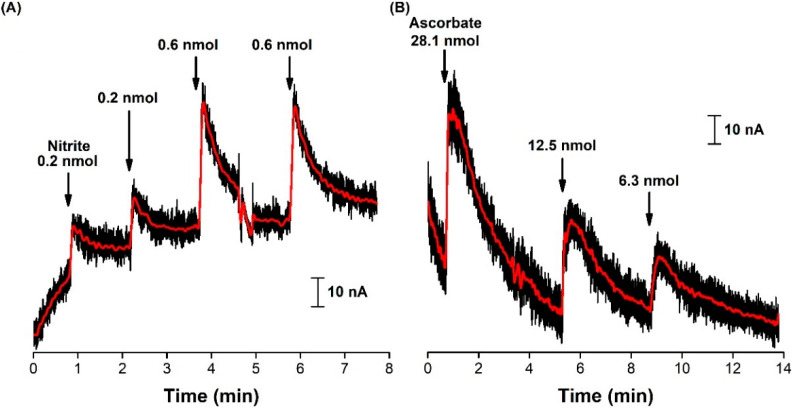
Measurements by FSCV of exogenously applied (**A**) nitrite and (**B**) ascorbate in the hippocampus CA1 subregion of the rat hippocampus. Current was sampled at 1.1 V and 0.2 V for nitrite and ascorbate, respectively. Red traces represent the 100-points FFT filtered current signals.

The current variations for a series of nitrite injections in the brain are shown in Figure 5A, as well for ascorbate in Figure 5B. As in the FSCV profile recordings, the current sampling procedure was affected by electrical noise interference, which caused the baseline to shift briefly. Nonetheless, upon achieving a stable baseline, the standard solution was injected. As previously observed in the FSCV profiles, the presence of nitrite in the vicinity of the surface of the microelectrode was accompanied by an increase in the recorded anodic current at 1.1 V, with reproducible transient signals (see Table 2).

For the duplicated injections of the nitrite standard, the maximum current (*I*_Max_) values were consistent, corresponding to an average peak concentration of 57.5 ± 0.7 µM and 129 ± 8 µM, with an average rise time of 6 ± 1 s (from the lowest to highest current value detected due to the oxidation of the analyte). The total duration of the signal for the 0.2 nmol injections was also consistent (*c.a.* 80 s), although this was not the case for the 0.6 nmol injections (53 vs. 118 s), possibly due to a shift in the baseline, as observed between 4.5 and 5 min. To the best of the authors’ knowledge, this was the first time that nitrite clearance was directly recorded in real time, in vivo and in situ in the brain. Classically, nitrite detection has been successfully achieved in the brain, via microdialysis sampling coupled with amperometric [45,47], spectrophotometric (Griess reaction) [44], and chemiluminescence [58] detection. Although these procedures were able to determine the cerebral nitrite concentrations, they lacked the temporal and spatial information needed to study the in situ dynamics of the targeted analyte. Although the methodology described here does not allow for the determination of the basal levels of nitrite, it can be useful in assessing local variations with high temporal and spatial resolution. For example, it enables the assessment of nitrite kinetics in the brain, after a systemic administration.

As for the detection of exogenously applied ascorbate, the sampling potential was shifted to 0.2 V and, upon injection of the stock solution, transient signals were observed with consistent rise times (average 22 ± 3 s) and total signal duration (average 233 ± 21 s). Ferreira and co-workers reported similar rise time values for endogenous ascorbate (about 25 s), upon glutamatergic stimulation, by local injection of 20 mM l-glutamate in the rat hippocampus [17]. As observed in Section 3.1, the carbon microelectrode was shown to be more sensitive towards nitrite than ascorbate, which explains why the injection of 12.5 nmol ascorbate produced a maximum current increase (35 nA) that was equivalent to the one recorded for 0.6 nmol of nitrite (36/39 nA).

The real-time in vivo detection of nitrite transient signals in the brain, upon localized pressure-ejection of the standard solution, was thus shown to be achievable by applying carbon-based microsensors combined with FSCV measurements. However, and as previously mentioned, the potential at which nitrite is oxidized at these carbon surfaces is susceptible to interference by ^●^NO. This could be solved by employing a self-reference carbon fiber electrode with a Nafion^®^-modified surface, which would repel negatively charged molecules (nitrite and ascorbate), but still be permeable to ^●^NO [36]. In this manner, the contribution of ^●^NO oxidation could be subtracted from the overall current measured at 1.1 V vs. Ag/AgCl. 

## 4. Conclusions

The importance of measuring nitrite in real-time in the brain extracellular space comes from the key interest in its putative role as a mediator of ^●^NO signaling, especially in conditions of diminished blood flow to the brain tissue, such as in hypoxic states. In this work, we studied the coupling of carbon fiber microelectrode-based sensors with FSCV and fixed potential sampling, for the real-time monitoring of nitrite in vivo. First, the microelectrodes’ response to this anion was characterized in vitro, in the presence of increasing physiological concentrations of ascorbate, which is a potential electrochemical interferent. Nitrite was effectively detected anodically in the 5–500 µM range, with negligible interference from ascorbate (only at 500 µM ascorbate, a small increase in the baseline current was observed). Then, the same sensing platform was able to measure fast transient nitrite oxidation signals in the hippocampus of an anesthetized rat, following the pressure ejection of exogenous nitrite into the brain tissue. These results demonstrate that the combined approach CFM/FSCV is a valuable tool to probe the nitrite’s temporal and spatial dynamics in vivo and in real-time, paving the way for a wide study on the role of nitrite in brain redox signaling and on its potential beneficial effects in neurovascular function.

## Figures and Tables

**Table 1:** Analytical parameters (as obtained by FSCV, at sample and hold potential 1.1 V vs. Ag/AgCl) of the carbon fiber microelectrode towards nitrite oxidation, in the presence of increasing concentrations of ascorbate. Measurements were performed in 50 mM phosphate buffer (pH 7.4), with 100 mM NaCl.

Nitrite Linear Range. (µM)	Ascorbate (µM)	CFM Analytical Parameters for Nitrite		
		Sens._Nitrite_ (pA M^−1^)	LOD_Nitrite_ (µM)	R^2^
5–500	0	135	2.7	0.999
5–500	100	124	9.2	0.998
5–500	200	119	8.4	0.998
5–500	500	107	5.2	0.999

**Table 2:** Parameters calculated from injections of nitrite and ascorbate in the CA1 subregion of the rat hippocampus, using a carbon fiber microsensor. The peaks’ concentrations were determined using the previously obtained calibration curves–nitrite: y_current_ = 331 pA/µM × x_[Nitrite]_ − 5 nA (R^2^ 0.999); ascorbate: y_current_ = 235 pA/µM × x_[Ascorbate]_ − 2 nA (R^2^ 0.996).

Amount of. Injected Species	*I*_Max_ (nA)	t_rise_ (s)	Signal Duration (s)	Concentration at Peak (µM)
**Nitrite (nmol)**				
0.2	14	5	82	57
0.2	14	4	81	58
0.6	39	6	53	135
0.6	36	8	118	123
**Ascorbate (nmol)**				
6.3	22	22	236	85
12.5	35	25	211	142
28.1	59	20	252	243

*I*_Max_—current at the signal’s maximum; t_rise_—signal rise time.

## Data Availability

The data presented in this study are available on request from the corresponding author.

## Supplementary Materials

The following are available online at https://www.mdpi.com/article/10.3390/bios11080277/s1, Figure S1: schematic representation of a homemade glass-encased carbon fiber microelectrode. The electrical contact between the fiber and the copper wire was made using conductive silver paint. Figure S2: schematic representation of (A) the animal in the stereotaxic frame and (B) the placement of the array used in the electrochemical recordings (microelectrode attached to an ejection micropipette) in the hippocampus (CA1 subregion). Adapted from [40].

## References

[1] Alderton W.K., Cooper C.E., Knowles R.G. (2001). Nitric oxide synthases: Structure, function and inhibition. Biochem. J..

[2] Hu Y., Zhu D. (2014). Hippocampus and Nitric Oxide. Vitamins and Hormones: Nitric Oxide.

[3] Liu H., Li J., Zhao F., Wang H., Qu Y., Mu D. (2015). Nitric oxide synthase in hypoxic or ischemic brain injury. Rev. Neurosci..

[4] Stuehr D.J. (2004). Enzymes of the L-Arginine to Nitric Oxide Pathway. J. Nutr..

[5] Jung J.E., Kim G.S., Chen H., Maier C.M., Narasimhan P., Song Y.S., Niizuma K., Katsu M., Okami N., Yoshioka H. (2010). Reperfusion and Neurovascular Dysfunction in Stroke: From Basic Mechanisms to Potential Strategies for Neuroprotection. Mol. Neurobiol..

[6] Lourenço C.F., Ledo A., Barbosa R.M., Laranjinha J. (2017). Neurovascular-neuroenergetic coupling axis in the brain: Master regulation by nitric oxide and consequences in aging and neurodegeneration. Free Radic. Biol. Med..

[7] Pereira C., Ferreira N.R., Rocha B.S., Barbosa R.M., Laranjinha J. (2013). The redox interplay between nitrite and nitric oxide: From the gut to the brain. Redox Biol..

[8] Lundberg J.O., Weitzberg E., Gladwin M.T. (2008). The nitrate-nitrite-nitric oxide pathway in physiology and therapeutics. Nat. Rev. Drug Discov..

[9] Bryan N.S., Fernandez B.O., Bauer S.M., Garcia-Saura M.F., Milsom A.B., Rassaf T., Maloney R.E., Bharti A., Rodriguez J., Feelisch M. (2005). Nitrite is a Signaling Molecule and Regulator of Gene Expression in Mammalian Tissues. Nat. Chem. Biol..

[10] Pinheiro L.C., Ferreira G.C., Damacena de Angelis C., Toledo J.C., Tanus-Santos J.E. (2020). A comprehensive time course study of tissue nitric oxide metabolites concentrations after oral nitrite administration. Free Radic. Biol. Med..

[11] Duncan C., Dougall H., Johnston P., Green S., Brogan R., Leifert C., Smith L., Golden M., Benjamin N. (1995). Chemical generation of nitric oxide in the mouth from the enterosalivary circulation of dietary nitrate. Nat. Med..

[12] Koch C.D., Gladwin M.T., Freeman B.A., Lundberg J.O., Weitzberg E., Morris A. (2017). Enterosalivary nitrate metabolism and the microbiome: Intersection of microbial metabolism, nitric oxide and diet in cardiac and pulmonary vascular health. Free Radic. Biol. Med..

[13] Lundberg J.O., Carlström M., Weitzberg E. (2018). Metabolic Effects of Dietary Nitrate in Health and Disease. Cell Metab..

[14] Piknova B., Kocharyan A., Schechter A.N., Silva A.C. (2011). The Role of Nitrite in Neurovascular Coupling. Brain Res..

[15] Millar J. (1995). The nitric oxide/ascorbate cycle: How neurones may control their own oxygen supply. Med. Hypotheses.

[16] Wilson J.X., Peters C.E., Sitar S.M., Daoust P., Gelb A.W. (2000). Glutamate stimulates ascorbate transport by astrocytes. Brain Res..

[17] Ferreira N.R., Lourenço C.F., Barbosa R.M., Laranjinha J. (2015). Coupling of ascorbate and nitric oxide dynamics in vivo in the rat hippocampus upon glutamatergic neuronal stimulation: A novel functional interplay. Brain Res. Bull..

[18] Dale N., Hatz S., Tian F., Llaudet E. (2005). Listening to the brain: Microelectrode biosensors for neurochemicals. Trends Biotechnol..

[19] Robinson D.L., Hermans A., Seipel A.T., Wightman R.M. (2008). Monitoring Rapid Chemical Communication in the Brain. Chem. Rev..

[20] Saldanha C., de Almeida J.P., Silva-Herdade A.S. (2014). Application of a Nitric Oxide Sensor in Biomedicine. Biosensors.

[21] Vieira D., McEachern F., Filippelli R., Dimentberg E., Harvey E.J., Merle G. (2020). Microelectrochemical Smart Needle for Real Time Minimally Invasive Oximetry. Biosensors.

[22] Zhang Z., Naughton D.P., Blake D.R., Benjamin N., Stevens C.R., Winyard P.G., Symone M.C.R., Harrison R. (1997). Human xanthine oxidase converts nitrite ions into nitric oxide (NO). Biochem. Soc. Trans..

[23] Cahill P.S., Walker Q.D., Finnegan J.M., Mickelson G.E., Travis E.R., Wightman R.M. (1996). Microelectrodes for the Measurement of Catecholamines in Biological Systems. Anal. Chem..

[24] Barbosa R.M., Lourenço C.F., Santos R.M., Pomerleau F., Huettl P., Gerhardt G.A., Laranjinha J. (2008). In Vivo Real-Time Measurement of Nitric Oxide in Anesthetized Rat Brain. Methods in Enzymology.

[25] Michael A.C., Borland L.M. (2007). Electrochemical Methods for Neuroscience.

[26] Huffman M.L., Venton B.J. (2009). Carbon-fiber microelectrodes for in vivo applications. Analyst.

[27] Roberts J.G., Sombers L.A. (2018). Fast-Scan Cyclic Voltammetry: Chemical Sensing in the Brain and Beyond. Anal. Chem..

[28] Singh Y.S., Sawarynski L.E., Dabiri P.D., Choi W.R., Andrews A.M. (2011). Head-to-Head Comparisons of Carbon Fiber Microelectrode Coatings for Sensitive and Selective Neurotransmitter Detection by Voltammetry. Anal. Chem..

[29] Kita J.M., Wightman R.M. (2008). Microelectrodes for studying neurobiology. Curr. Opin. Chem. Biol..

[30] Ou Y., Buchanan A.M., Witt C.E., Hashemi P. (2019). Frontiers in electrochemical sensors for neurotransmitter detection: Towards measuring neurotransmitters as chemical diagnostics for brain disorders. Anal. Methods.

[31] Tan C., Robbins E.M., Wu B., Cui X.T. (2021). Recent Advances in In Vivo Neurochemical Monitoring. Micromachines.

[32] Garris P.A., Wightman R., Boulton A.A., Baker G.B., Adams R.N. (1995). Regional Differences in Dopamine Release, Uptake, and Diffusion Measured by Fast-Scan Cyclic Voltammetry BT—Voltammetric Methods in Brain Systems. Neuromethods.

[33] Si B., Song E. (2018). Recent Advances in the Detection of Neurotransmitters. Chemosensors.

[34] Brown F.O., Finnerty N.J., Lowry J.P. (2009). Nitric oxide monitoring in brain extracellular fluid: Characterisation of Nafion®-modified Pt electrodes in vitro and in vivo. Analyst.

[35] Ledo A., Barbosa R.M., Frade J., Laranjinha J. (2002). Nitric oxide monitoring in hippocampal brain slices using electrochemical methods. Methods Enzymol..

[36] Santos R.M., Lourenço C.F., Piedade A.P., Andrews R., Pomerleau F., Huettl P., Gerhardt G.A., Laranjinha J., Barbosa R.M. (2008). A comparative study of carbon fiber-based microelectrodes for the measurement of nitric oxide in brain tissue. Biosens. Bioelectron..

[37] Fourmond V. (2016). QSoas, A Versatile Software for Data Analysis. Anal. Chem..

[38] Lourenço C.F., Santos R., Barbosa R.M., Gerhardt G., Cadenas E., Laranjinha J. (2011). In vivo modulation of nitric oxide concentration dynamics upon glutamatergic neuronal activation in the hippocampus. Hippocampus.

[39] Ledo A., Lourenço C.F., Laranjinha J., Brett C.M.A., Gerhardt G.A., Barbosa R.M. (2017). Ceramic-Based Multisite Platinum Microelectrode Arrays: Morphological Characteristics and Electrochemical Performance for Extracellular Oxygen Measurements in Brain Tissue. Anal. Chem..

[40] Paxinos G., Watson C. (2007). The Rat Brain in Stereotaxic Coordinates.

[41] Zheng D., Hu C., Peng Y., Hu S. (2009). A carbon nanotube/polyvanillin composite film as an electrocatalyst for the electrochemical oxidation of nitrite and its application as a nitrite sensor. Electrochim. Acta.

[42] Lee W.H., Wahman D.G., Pressman J.G. (2013). Amperometric carbon fiber nitrite microsensor for in situ biofilm monitoring. Sens. Actuators B Chem..

[43] Piela B., Wrona P.K. (2002). Oxidation of Nitrites on Solid Electrodes: I. Determination of the Reaction Mechanism on the Pure Electrode Surface. J. Electrochem. Soc..

[44] Shibata M., Araki N., Hamada J., Sasaki T., Shimazu K., Fukuuchi Y. (1996). Brain nitrite production during global ischemia and reperfusion: An in vivo microdialysis study. Brain Res..

[45] Mao L., Shi G., Tian Y., Liu H., Jin L., Yamamoto K., Tao S., Jin J. (1998). A novel thin-layer amperometric detector based on chemically modified ring-disc electrode and its application for simultaneous measurements of nitric oxide and nitrite in rat brain combined with in vivo microdialysis. Talanta.

[46] Rizzo V., Montalbetti L., Rozza A., Bolzani W., Porta C., Balduzzi G., Scoglio E., Moratti R. (1998). Nitrite/nitrate balance during photoinduced cerebral ischemia in the rat determined by high-performance liquid chromatography with UV and electrochemical detection. J. Chromatogr. A.

[47] Sun W., Zhang S., Lin X., Jin L., Jin S., Deng J., Kong J. (1999). Electrocatalytic reduction of nitrite at a carbon fiber microelectrode chemically modified by palladium(II)-substituted Dawson type heptadecatungstodiphosphate. J. Electroanal. Chem..

[48] Woitzik J., Abromeit N., Schaefer F. (2001). Measurement of nitric oxide metabolites in brain microdialysates by a sensitive fluorometric high-performance liquid chromatography assay. Anal. Biochem..

[49] Gao L., Barber-Singh J., Kottegoda S., Wirtshafter D., Shippy S.A. (2004). Determination of nitrate and nitrite in rat brain perfusates by capillary electrophoresis. Electrophoresis.

[50] Iravani M.M., Millar J., Kruk Z.L. (2002). Differential Release of Dopamine by Nitric Oxide in Subregions of Rat Caudate Putamen Slices. J. Neurochem..

[51] Ferreira N.R., Ledo A., Laranjinha J., Gerhardt G.A., Barbosa R.M. (2018). Simultaneous measurements of ascorbate and glutamate in vivo in the rat brain using carbon fiber nanocomposite sensors and microbiosensor arrays. Bioelectrochemistry.

[52] Liu A., Honma I., Zhou H. (2007). Simultaneous voltammetric detection of dopamine and uric acid at their physiological level in the presence of ascorbic acid using poly(acrylic acid)-multiwalled carbon-nanotube composite-covered glassy-carbon electrode. Biosens. Bioelectron..

[53] Raj C.R., Ohsaka T. (2001). Electroanalysis of ascorbate and dopamine at a gold electrode modified with a positively charged self-assembled monolayer. J. Electroanal. Chem..

[54] Zhang M., Liu K., Xiang L., Lin Y., Su L., Mao L. (2007). Carbon Nanotube-Modified Carbon Fiber Microelectrodes for In Vivo Voltammetric Measurement of Ascorbic Acid in Rat Brain. Anal. Chem..

[55] Nemet I., Monnier V.M. (2011). Vitamin C Degradation Products and Pathways in the Human Lens. J. Biol. Chem..

[56] Rice M.E. (2000). Ascorbate regulation and its neuroprotective role in the brain. Trends Neurosci..

[57] May J.M., Stanger O. (2012). Vitamin C Transport and Its Role in the Central Nervous System. Subcellular Biochemistry.

[58] Goodman J.C., Feng Y.-Q., Valadka A.B., Bryan R.J., Robertson C.S. (2002). Measurement of the Nitric Oxide Metabolites Nitrate and Nitrite in the Human Brain by Microdialysis. Intracranial Pressure and Brain Biochemical Monitoring.

