# Supplementary material for: Microelectrode Sensor for Real-Time Measurements of Nitrite in the Living Brain, in the Presence of Ascorbate
Source: Biosensors (Basel). 2021 Aug 17;11(8):277. doi: 10.3390/bios11080277 (PMC8394717; doi:10.3390/bios11080277)
Supplement: Supplementary file 1 [file biosensors-11-00277-s001.zip › biosensors-1312192-supplementary.pdf]

Supplementary Materials

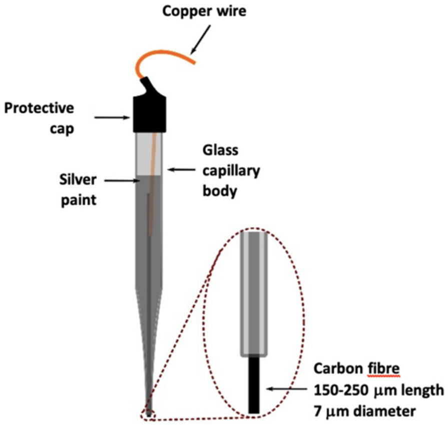

Figure S1. Schematic representation of a homemade glass-encased carbon fiber microelectrode. The electrical contact between the fiber and the copper wire was made using conductive silver paint.

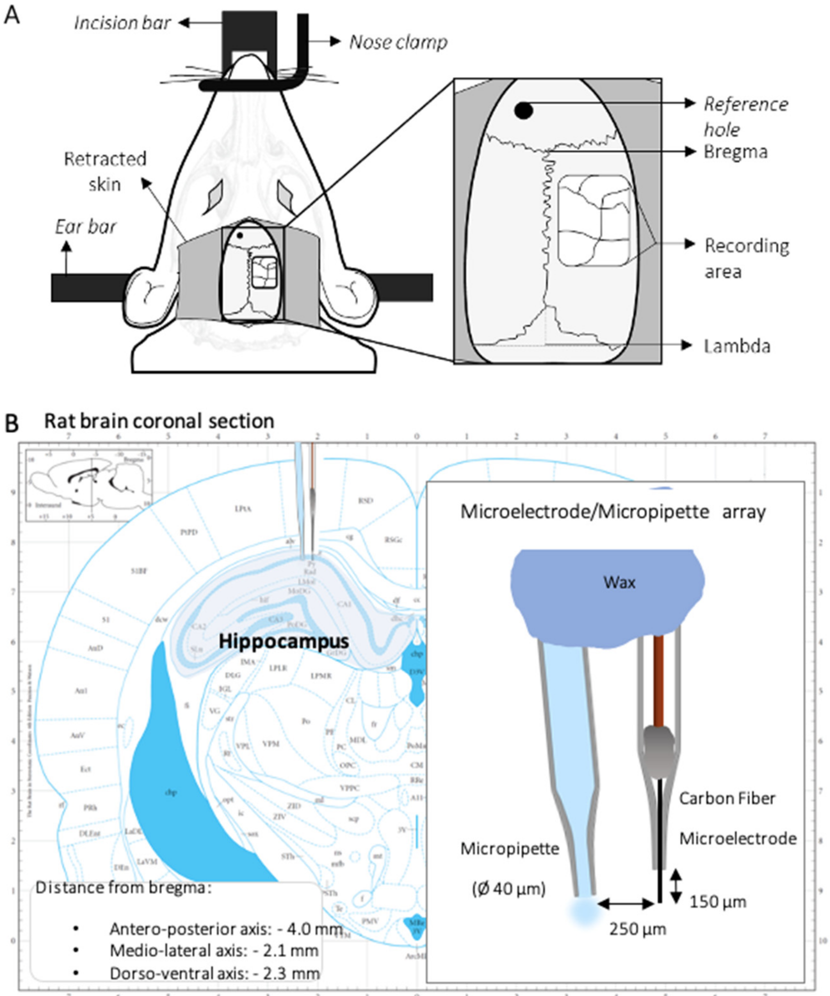

Figure S2. Schematic representation of (**A**) the animal in the stereotaxic frame and (**B**) the placement of the array used in the electrochemical recordings (microelectrode attached to an ejection micropipette) in the hippocampus (CA1 subregion). Adapted with permission from ref. [40]. 2007 Elsevier.
